# RepViz: a replicate-driven R tool for visualizing genomic regions

**DOI:** 10.1186/s13104-019-4473-z

**Published:** 2019-07-19

**Authors:** Thomas Faux, Kalle T. Rytkönen, Asta Laiho, Laura L. Elo

**Affiliations:** 1 Turku Bioscience Centre, University of Turku and Åbo Akademi University, Tykistökatu 6, 20520 Turku, Finland; 2 Institute of Biomedicine, Research Centre for Integrative Physiology and Pharmacology, University of Turku, Kiinamyllynkatu 10, 20014 Turku, Finland

**Keywords:** Visualisation, Genome analysis, Computational genomics, Epigenetics

## Abstract

**Objective:**

Visualization of sequencing data is an integral part of genomic data analysis. Although there are several tools to visualize sequencing data on genomic regions, they do not offer user-friendly ways to view simultaneously different groups of replicates. To address this need, we developed a tool that allows efficient viewing of both intra- and intergroup variation of sequencing counts on a genomic region, as well as their comparison to the output of user selected analysis methods, such as peak calling.

**Results:**

We present an R package RepViz for replicate-driven visualization of genomic regions. With ChIP-seq and ATAC-seq data we demonstrate its potential to aid visual inspection involved in the evaluation of normalization, outlier behavior, detected features from differential peak calling analysis, and combined analysis of multiple data types. RepViz is readily available on Bioconductor (https://www.bioconductor.org/packages/devel/bioc/html/RepViz.html) and on Github (https://github.com/elolab/RepViz).

**Electronic supplementary material:**

The online version of this article (10.1186/s13104-019-4473-z) contains supplementary material, which is available to authorized users.

## Introduction

DNA-sequencing has become an essential part of biomedicine and biology. Several computational tools have been developed for analyzing such data. However, a visual inspection of the data by a researcher is still important both at the level of basic quality control and as a confirmation of the analysis results. Visualization can also guide the analysis design and interpretation of the results. Numerous tools have been developed to visualize genomic data, including UCSC genome browser [1], Integrative Genomics Viewer (IGV) [2], or BamView [3]. Additional tools are available in R such as ggbio [4], GenVisR [5], Gviz [6], rbamtools [7], Sushi [8]. Other R tools like Genomation [9] and ChIPpeakAnno [10] enable the visualization of the genome by taking the average of multiple regions or via a heatmap, but lack the resolution of read coverage. Altogether, there is still a demand for a specific tool to efficiently visualize groups of biological replicates at specific genomic locus.

Currently, genomic visualization of the sequencing data is especially important in the analysis of chromatin data, such as ChIP-seq and ATAC-seq. Specific histone modification markers with distinct dynamics require custom parameterization in calling the differential signal and, therefore, constitute a more complex situation compared to, for example, RNA-seq analysis [11, 12]. Accordingly, the selection of a proper peak calling or differential peak calling tool and parameters for specific histone modification markers is often a complex and iterative process in which visualization has an important role. Visualization of the intragroup replicates can be used to check if the assumptions of a given differential peak caller are met with the analyzed data. Additionally, visualization of the replicates can guide the evaluation of the normalization steps [13, 14] and identification of potential outliers.

In an effort to provide a user-friendly tool to visualize groups of replicates on genomic regions, we propose a replicate-driven R tool, RepViz. RepViz allows simultaneous viewing of both intra- and intergroup variation in sequencing counts of the studied conditions, as well as their comparison to the output features (e.g. identified peaks) from user selected analysis methods. The RepViz tool is primarily designed for chromatin data, such as ChIP-seq and ATAC-seq, but can also be used with other sequencing data, such as RNA-seq, or combinations of different types of genomic data.

### Implementation

**Fig. 1:**
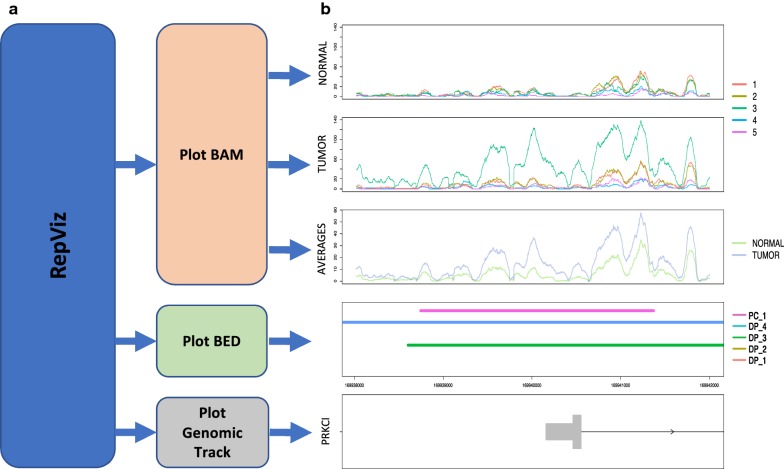
Overview and biological case examples of RepViz using histone modification data. **a** Overview of the main functions implemented in the software. **b** Example of a promoter region marked by H3K27Ac (GSE85467), where the mean signal in the second biological condition is driven by a single outlier replicate, but still leads to a significant differential peak call by multiple differential peak callers. Five replicates in two biological conditions are displayed in the first two panels and their mean signals in the third panel. The fourth panel is a visualization of the called peaks, where the uppermost row is from a peak caller MACS2 (PC) and the other rows are from differential peak callers (DP) (Additional file 1: Table S2). The lowest panel is the genomic track

RepViz is implemented in R and can run on both MacOS, Windows, and Linux. The tool uses comma-separated value (CSV) files as an input and is easy to use. RepViz is divided into three main functions that produce the visual outputs (Fig. 1a). The first function visualizes Binary Alignment Map (BAM) data. In the visualization, the samples are organized by group and the different replicates are color-coded; an additional visualization is produced for the group averages (Fig. 1b, three upper panels). The second function enables the visualization of Browser-Extensible Data (BED) files, such as peaks detected by a peak calling software. This enables software comparison or replicate comparison after individual peak calling (Fig. 1b, fourth panel). The third function is for visualizing the genomic track. The default input consists of two CSV files: one related to the BAM files and another optional file related to the BED files (Fig. 1b, lower panel).

## Main text

### Data processing, peak calling and differential peak calling

We tested RepViz with public data from GEO and using available tools for peak calling and differential peak calling. Details of the sequencing data used in the examples are provided in Additional file 1: Table S1, and details of the peak caller and differential peak callers are provided in Additional file 1: Table S2. The quality of the sequencing data was assessed with FastQC (http://www.bioinformatics.babraham.ac.uk/projects/fastqc) and the fastq files were aligned against reference genome (mm10 and hg19 according to cases) with Bowtie 2 (2.2.6) [15]. The peaks were called using MACS2 (2.1.1) [16] with the parameters–*broad*–*nomodel* -*q 0.05.* The differential peak callers can be roughly divided in two categories: the one step methods (PePr [17], THOR [14] and diffReps [18]) that use their own peak callers and the two step method (DiffBind [19]) that requires an external peak caller. For DiffBind we used the peaks called with MACS2. The differential peak calling was done with the default settings of the software cited in Additional file 1: Table S2. To emphasize that the scope of this study is the visualization tool the differential peak callers were randomly numbered in the examples.

### Results and discussion

Our R tool, RepViz, enables the user to take a snapshot of a defined genomic region with multiple data inputs and visualize it in an efficient manner. Unlike the commonly used visualization tools, it implements a replicate-driven approach, allowing user-friendly visualization of replicates within and between experimental conditions. Here we provide examples on how RepViz can aid visual inspection involved in the evaluation of outlier behavior, normalization, differential peak calling analysis and combined analysis of multiple data types. Details of the sequencing data, peak calling and differential peak calling used in the examples are provided in Additional file 1.

**Fig. 2:**
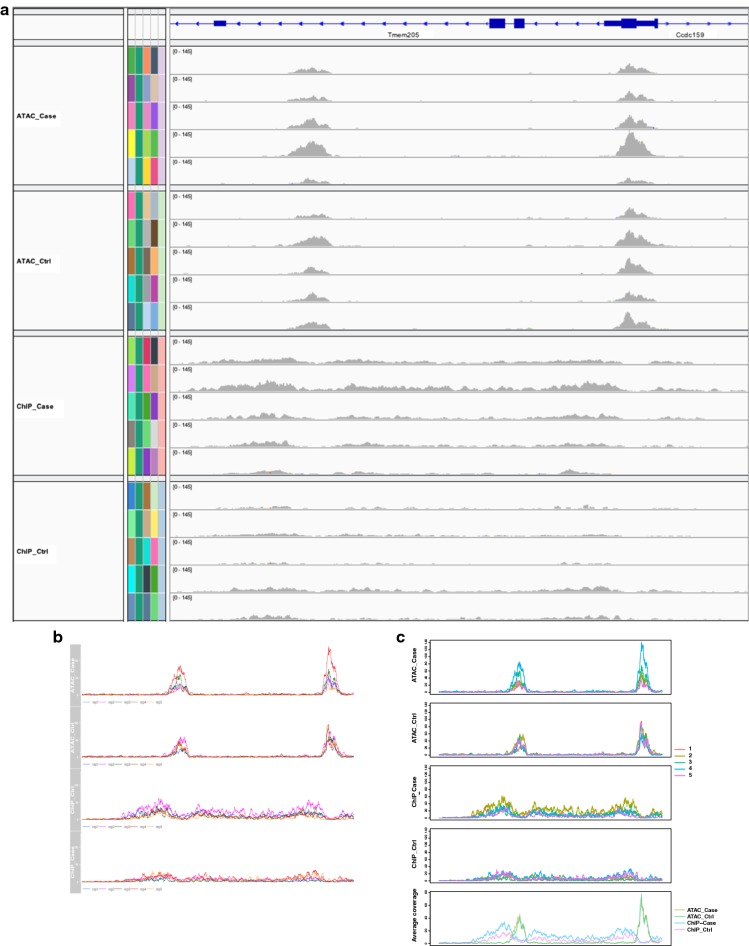
Comparison of (**a**) IGV, (**b**) Gviz, and (**c**) RepViz. IGV does not allow grouping of replicates to be presented in one track. With Gviz showing replicates in one track is possible, but it is not possible to compare a different number of grouped replicates to each other or include the average coverage under the grouped tracks like in RepViz. Data from GSE108990. Panels from the top to bottom are ATAC-seq case, ATAC-seq control, ChIP-seq case, and ChIP-seq control. In (**c**) the lower panel is representing the group-wise average signals for each of the conditions

The first function of RepViz visualizes BAM files by presenting all the replicates on the same scale as well as their group-wise averages. This can be used to assess the similarity between the replicates within a given biological condition, or if the average signal is affected by outliers (Fig. 1b). The replicate-driven visualization is also a useful confirmatory step for normalization, enabling for instance, comparison of replicates after normalization at known house-keeping genes (Additional file 1: Fig. S1). With the current genomic browsers, this type of visualization can be a time-consuming task. For instance, IGV does not have an option to group tracks leading to the replicates being stacked on top of each other, whereas Gviz has an option to group samples together but does not allow comparing groups with a different number of grouped replicates (see Fig. 2 for more details of the comparison).

The second function of RepViz visualizes multiple BED files, which can help, for instance, to compare different peak calling software. By comparing the called peaks to the observed data for each replicate (BAM) the user can visually confirm the called features (Fig. 1b, Additional file 1: Fig. S2). For example, in the case of ChIP-seq studies, differential peak calls can be easily inspected in the light of replicate behavior, and peak calls that are driven by outliers can be detected (Fig. 1b). Additionally, the tool allows a replicate-driven inspection of the length of the called peak. This is useful because several peak callers tend to combine clusters of sharp peaks to broader peaks [11, 12]. Finally, the third function of RepViz visualizes the gene track to display the genes in the region of interest, such as gene promoters or their vicinity.

**Fig. 3:**
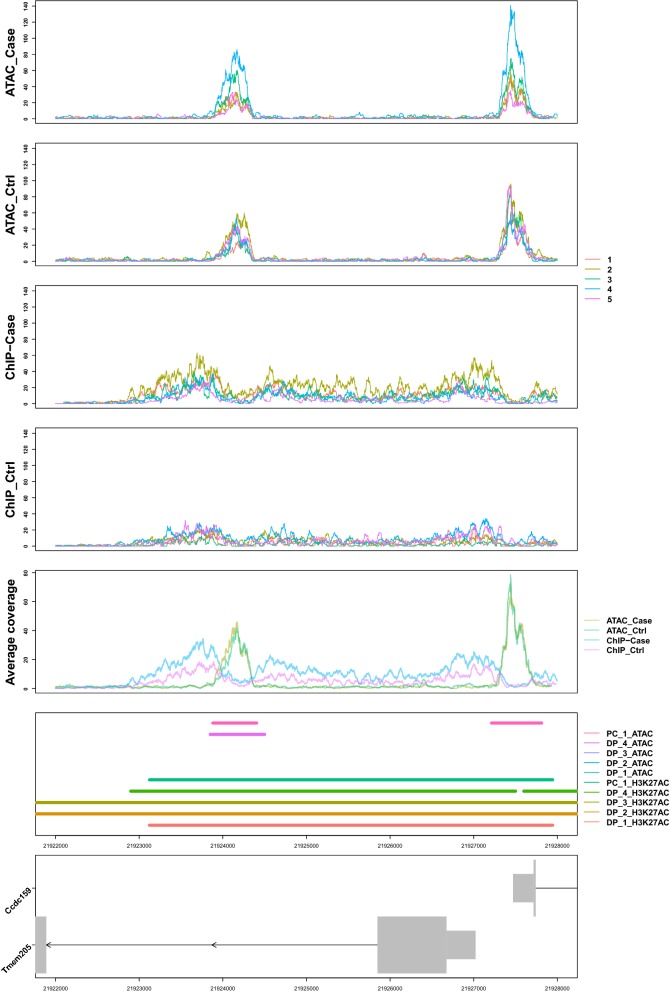
Example of a combined visualization of ATAC-seq and H3K27ac histone modification marker using replicate matched data. *PC* peak caller, *DP* differential peak caller. Data from GSE108990

In addition to visualizing replicates within a particular data type, RepViz can visualize multiple data types (datasets) simultaneously by considering each dataset as a separate group in the input file. With multiple matched datasets, the replicate-driven visual inspection can be useful for both evaluating the quality of the samples as well as assessing the performance of the differential peak calling methods between datasets with different dynamics (Additional file 1: Fig. S3). Moreover, a combined visualization of matched histone marker and ATAC-seq data can provide replicate specific insights for the relationship of histone modification and open chromatin state (Fig. 3). Other potential applications of RepViz include, for example, the combination of chromatin marker or ATAC-seq data with eRNA [20] or non-coding RNA data to inspect replicate variability on chromatin level together with RNA expression variability at specific genomic regions. RepViz will be actively maintained and further developed.

## Limitations

RepViz has been developed in order to get a quick snapshot of a genomic region. Large genomic regions can be slow to print. While ready on the user end, the efficiency of the code can still be improved in the later versions. RepViz has initially been thought for user with minimal knowledge in R, it will be developed in a more advanced user-friendly manner later on.

## Additional file

**Additional file 1.** The file contains additional tables and additional figures.

## Data Availability

The datasets supporting the conclusions of this article are available in the Gene Expression Omnibus data-base, [Accession Number: GSE85467; https://www.ncbi.nlm.nih.gov/geo/query/acc.cgi?acc=GSE85467, GSE108990; https://www.ncbi.nlm.nih.gov/geo/query/acc.cgi?acc=GSE108990]. *Software information:* Project Name: RepViz. Project home page: http://bioconductor.org/packages/devel/bioc/html/RepViz.html. Archived version: v1.0.0. Operating system(s): Platform independent. Programming language: R. Other Requirements: Not Applicable. License: GPL-3.

## Abbreviations

BAM: binary alignment map
BED: browser extensible data
CSV: coma-separated value
DNA: deoxyribonucleic acid
RNA: ribonucleic acid
eRNA: enhancer RNA
ATAC-seq: assay for transposable accessible chromatin sequencing
ChIP-seq: chromatin immuno-precipitation sequencing
RNA-seq: RNA sequencing
