# Supplementary material for: RepViz: a replicate-driven R tool for visualizing genomic regions
Source: BMC Res Notes. 2019 Jul 19;12:441. doi: 10.1186/s13104-019-4473-z (PMC6642542; doi:10.1186/s13104-019-4473-z)
Supplement: Supplementary file 1 — Additional file 1. The file contains additional tables and additional figures. [file 13104_2019_4473_MOESM1_ESM.docx]

**Additional Data**

**RepViz: a replicate-driven R tool for visualizing genomic regions**

Thomas Faux^1^, Kalle T. Rytkönen^1,2^, Asta Laiho^1^, Laura L. Elo^1^

^1^Turku Bioscience Centre, University of Turku and Åbo Akademi University, Tykistökatu 6, FI-20520, Turku, Finland. ^2^Institute of Biomedicine, Research Centre for Integrative Physiology and Pharmacology, University of Turku, Kiinamyllynkatu 10, FI-20014, Finland.

**Additional tables**

**Table S1.** The sequencing data used in the application examples.

| **Data** | **Accession** | **Reference** |
| --- | --- | --- |
| ChIP-seq (H3K27Ac) | GSE85467 | [2] |
| ChIP-seq (H3K4Me3) | GSE85467 | [2] |
| ATAC-seq  ChIP-seq (H3K27Ac) | GSE108990  GSE108990 | [3]  [3] |

**Table S2.** The software used in the application examples. MACS2 is a peak caller and others are differential peak callers.

| **Software** | **Reference** |
| --- | --- |
| DiffBind | [4] |
| diffReps | [5] |
| PePr | [6] |
| THOR | [7] |
| MACS2.1 (peak caller) | [8] |

**Additional figures**

**A**

**B**

**Fig. S1.** Example comparing (A) ATAC-seq open chromatin and (B) H3K27ac histone modification marker chromatin occupancy between replicates to see if depth normalized replicates have similar mean intensities between the conditions at the house-keeping gene EMC7 locus [9]. DP = differential peak caller. Data from GSE108990.

**Fig. S2.** Example of a promoter region where multiple peak calling tools detect significant differential H3K27ac chromatin occupancy and there are no outliers in the replicate groups. PC = peak caller, DP = differential peak caller. Data from GSE85467.

**Fig. S3.** Example of a combined visualization of multiple ChIP-seq datasets, including the visualization of the same H3K27ac data and region as in Fig. 1B with replicate matched H3K4me3 data. The BED files from differential peak callers are shown for both the H3K27ac and the H3K4me3 data**.** In the case of H3K27ac, the outlier with high read density leads to a differential peak call with two tools (DP_3 and DP_5), whereas with H3K4me3 the moderate outlier leads to a differential peak call with one tool (DP_2). PC = peak caller, DP = differential peak caller. Data from GSE85467.

**References**

1. Langmead B, Salzberg SL. Fast gapped-read alignment with Bowtie 2. Nat Methods. 2012;9:357–9.

2. Ooi WF, Xing M, Xu C, Yao X, Ramlee MK, Lim MC, et al. Epigenomic profiling of primary gastric adenocarcinoma reveals super-enhancer heterogeneity. Nat Commun. 2016;7:12983.

3. Israel JW, Chappell GA, Simon JM, Pott S, Safi A, Lewis L, et al. Tissue- and strain-specific effects of a genotoxic carcinogen 1,3-butadiene on chromatin and transcription. Mamm Genome. 2018;29:153–67.

4. Ross-Innes CS, Stark R, Teschendorff AE, Holmes KA, Ali HR, Dunning MJ, et al. Differential oestrogen receptor binding is associated with clinical outcome in breast cancer. Nature. 2012;481:389–93.

5. Shen L, Shao N-Y, Liu X, Maze I, Feng J, Nestler EJ. diffReps: Detecting Differential Chromatin Modification Sites from ChIP-seq Data with Biological Replicates. PLoS One. 2013;8:e65598.

6. Zhang Y, Lin Y-H, Johnson TD, Rozek LS, Sartor MA. PePr: a peak-calling prioritization pipeline to identify consistent or differential peaks from replicated ChIP-Seq data. Bioinformatics. 2014;30:2568–75.

7. Allhoff M, Seré K, F. Pires J, Zenke M, G. Costa I. Differential peak calling of ChIP-seq signals with replicates with THOR. Nucleic Acids Res. 2016;44:gkw680. doi:10.1093/nar/gkw680.

8. Zhang Y, Liu T, Meyer CA, Eeckhoute J, Johnson DS, Bernstein BE, et al. Model-based Analysis of ChIP-Seq (MACS). Genome Biol. 2008;9:R137.

9. Eisenberg E, Levanon EY. Human housekeeping genes, revisited. Trends Genet. 2013;29:569–74.
